# A Triaxial Applicator for the Measurement of the Electromagnetic Properties of Materials

**DOI:** 10.3390/s18020383

**Published:** 2018-01-29

**Authors:** Saranraj Karuppuswami, Edward Rothwell, Premjeet Chahal, Michael Havrilla

**Affiliations:** 1 Department of Electrical and Computer Engineering, Michigan State University, East Lansing, MI 48824, USA; karuppus@msu.edu (S.K.); chahal@egr.msu.edu (P.C.); 2 Department of Electrical and Computer Engineering, Air Force Institute of Technology, Wright-Patterson Air Force Base, OH 45433-7765, USA; michael.havrilla@afit.edu

**Keywords:** permeability, permittivity, material characterization, microwave measurements, S-parameters, calibration, two-port networks, electromagnetic analysis

## Abstract

The design, analysis, and fabrication of a prototype triaxial applicator is described. The applicator provides both reflected and transmitted signals that can be used to characterize the electromagnetic properties of materials in situ. A method for calibrating the probe is outlined and validated using simulated data. Fabrication of the probe is discussed, and measured data for typical absorbing materials and for the probe situated in air are presented. The simulations and measurements suggest that the probe should be useful for measuring the properties of common radar absorbing materials under usual in situ conditions.

## 1. Introduction

Magnetic radar absorbing materials (MagRAMs) are often applied to the surfaces of aircraft to reduce radar cross-section. They are generally formulated by suspending magnetic particles in an elastomer [1]. The resulting properties are highly dependent on filling factor and processing techniques [2], and thus the electromagnetic properties of the materials must be verified using experimental techniques. Fortunately, a wide variety of techniques have been devised to measure both the permeability and permittivity of material samples [3]. However, many of these require the insertion of a sample into a field applicator and are thus destructive methods useful only in the laboratory [4,5,6,7,8,9,10].

Because the electromagnetic properties of the materials degrade with age and exposure to the environment, it is important to regularly verify their performance without removing them from the aircraft. This requires non-destructive techniques to measure the complex permittivity ϵ and complex permeability μ of these microwave absorbers in situ. Because two properties are desired, two sufficiently different complex measurements must be made. However, when a material’s coating is directly adhered to an aircraft’s surface, the presence of the conductor backing prevents the measurement of a directly transmitted wave, and reflection/transmission techniques such as those presented in [4,5] cannot be used. Specialized techniques that use knowledge of the material’s constituency are possible [11], but usually two different reflection measurements are employed [12]. Unfortunately, the restrictions imposed by in situ measurements exclude such otherwise useful techniques as the layer-shift method [13] and the two-thickness method [14], while free-space techniques that depend on varying the angle or polarization are generally unreliable [12]. Similarly, techniques that rely on a single open-ended probe [15,16,17,18] have not proven to be sufficiently robust.

Systems that utilize a transmitted signal without needing access to both sides of the sample have been devised. These generally involve the propagation of a wave through the sample between two applicators placed on the open surface of the material. In [19], a microstrip transmission line is used to guide the wave from one measurement port to another to provide the transmitted signal, while in [20,21], two adjacent rectangular waveguide probes are placed against the material’s surface. In the case of the waveguide probes, the signals reflected at each probe and the signal transmitted between the probes are measured using a vector network analyzer (VNA). By comparing the measured signals to those predicted from a full-wave numerical solution, the material’s properties may be retrieved.

The dual-waveguide probe system shows great promise, but suffers from two deficiencies. First, as a result of the orientation of the waveguide fields, the strength of the transmitted signal is usually quite low, resulting in sensitivity to noise and measurement errors. Second, the complexity of the rectangular probes results in a difficult and time-consuming full-wave solution, which must be performed iteratively during the search that determines the material’s properties.

An alternative to the dual-rectangular-waveguide probe system was proposed in [22]. A triaxial system consisting of two coaxial transmission lines, one centered within the other, is placed directly against the material’s coating. As with the dual-waveguide system, the signal transmitted between the coaxial apertures and the signals reflected from the apertures may be used to determine ϵ and μ . However, the triaxial applicator has several advantages over the dual-waveguide probe. Because the guiding structures are transmission lines, the bandwidth of the system is potentially large (limited in practice by the onset of higher-order modes in the larger coax). Additionally, the transmission between the two coaxial lines, through the sample, is reasonably strong across much of the operating band. Lastly, the azimuthal symmetry of the triaxial system greatly reduces the complexity of the full-wave solution to the theoretical problem.

The solution for a simple model of the triaxial system was outlined in [22], providing the theoretical S-parameters of the two-port system describing the transverse electromagnetic (TEM)-mode behavior of the waves at the material’s plane. However, no practical implementation of the structure was proposed. There are two difficulties involved in producing a practical working triaxial probe. First, a transition between a coaxial line and the outer coax of the triaxial system must be devised, in which the outer conductors of both the inner and outer coaxial cables of the triax are held at ground potential. This is important to protect the receiver inputs of the VNA, which are sensitive to static discharge. Second, a means of calibrating the system to remove the effects of the transitions must be established. This paper presents a working prototype of a triaxial probe system. The geometry of the probe is described, and a brief outline of the theoretical model and its solution are given. The extraction procedure, based on comparing the measured and theoretical values of the S-parameters, is outlined. This procedure requires the S-parameters at the material’s plane, and thus a calibration process is described to de-embed the material plane’s S-parameters from the S-parameters measured at the ports where the VNA cables are connected. Finally, the construction of a working prototype is discussed, and measurements of absorbing material samples and of the triaxial system situated in air are given. The results for air are also compared to simulations using the High-Frequency Structure Simulator (HFSS) commercial solver.

## 2. Probe Geometry

**Figure 1:**
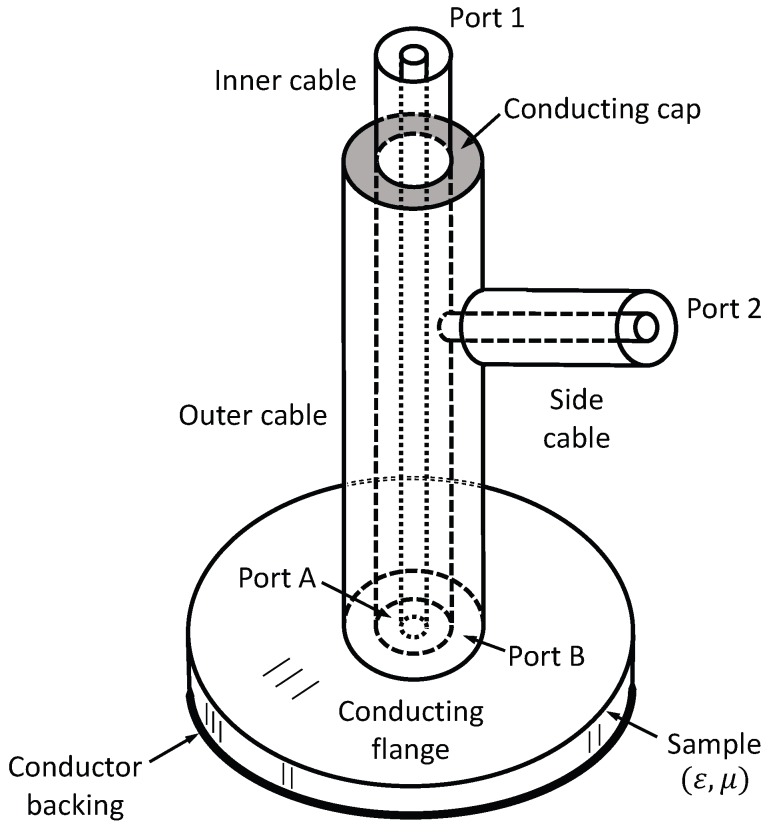
Geometry of the triaxial applicator.

Figure 1 shows the geometry of the triaxial probe system. The two concentric coaxial cables terminate in a circular metal flange that is placed against the surface of a planar, conductor-backed material sample. Because the materials of interest are absorbing, the flange radius $r_{F}$ must only be large enough such that cylindrical waves propagating outward from the coaxial cable openings in the material’s region between the flange and the conductor backing are sufficiently attenuated to render them insignificant upon reflection at the flange edge. It is possible to use a smaller flange, or to use lower-loss materials, if time-gating is employed [23]. This approach may be considered in future implementations of the system.

The inner coaxial cable of the triax extends directly from the material’s Port A to the measurement Port 1, where one test-port cable of the VNA is attached. The outer coaxial cable extends from the material’s Port B to a conducting cap that acts as a short-circuit termination. In this way, the potential of the outer conductor of the inner cable and the outer conductor of the outer cable, along with the flange, are held at ground potential. This protects the inputs to the VNA from damage due to static discharge when touching the structure. The outer coax is excited through a side cable of the same size as the inner cable. The outer conductor of the side cable connects to the outer conductor of the outer coaxial cable and is thus at ground potential. The inner conductor of the side cable extends through an aperture in the outer cable wall and is physically connected to the outer conductor of the inner cable. This side cable extends outward to the measurement Port 2, where the second test-port cable of the VNA is attached. Thus, the measured S-parameters are the reflection coefficient at Port 1, $S_{11}$ ; the reflection coefficient at Port 2, $S_{22}$ ; and the transmission coefficients between the measurement ports, $S_{21}=S_{12}$ . By using the calibration procedure described below, the reflection coefficients for the dominant TEM mode at the material’s plane, $S_{11}^{M}$ and $S_{22}^{M}$ , and the transmission coefficients of the TEM mode at the material’s plane $S_{21}^{M}=S_{12}^{M}$ , may be de-embedded. The de-embedded parameters are then used to determine the desired material parameters, as described in the next section.

## 3. Theoretical Model and Extraction Procedure

In order to extract the material parameters μ and ϵ , a theoretical model is required that produces S-parameters that may be compared to measurement. We let $S_{11}^{M}$ be the measured reflection coefficient for the inner coax at the material’s plane (Port A), as determined by the calibration procedure described below. Similarly, we let $S_{22}^{M}$ be the measured reflection coefficient for the outer coax at the material’s plane (Port B), as determined by the calibration procedure, and $S_{21}^{M}$ be the measured transmission coefficient at the material’s plane (from Port A to Port B). The permeability μ and permittivity ϵ of the material under test may then be found by solving the simultaneous complex equations:

(1) $$\begin{array}{c} S_{11}^{\mathrm{THY}}\left(\epsilon ,\mu \right)-S_{11}^{M}=0 \end{array}$$

(2) $$\begin{array}{c} S_{21}^{\mathrm{THY}}\left(\epsilon ,\mu \right)-S_{21}^{M}=0 \end{array}$$

at each frequency. Here $S_{11}^{\mathrm{THY}}$ and $S_{21}^{\mathrm{THY}}$ are the reflection and transmission coefficients at the material’s plane, determined by a theoretical solution to a model of the triax system. We note that $S_{22}$ could be used in place of $S_{11}$ , or both $S_{11}$ and $S_{22}$ could be used and a least-squares solution sought.

**Figure 2:**
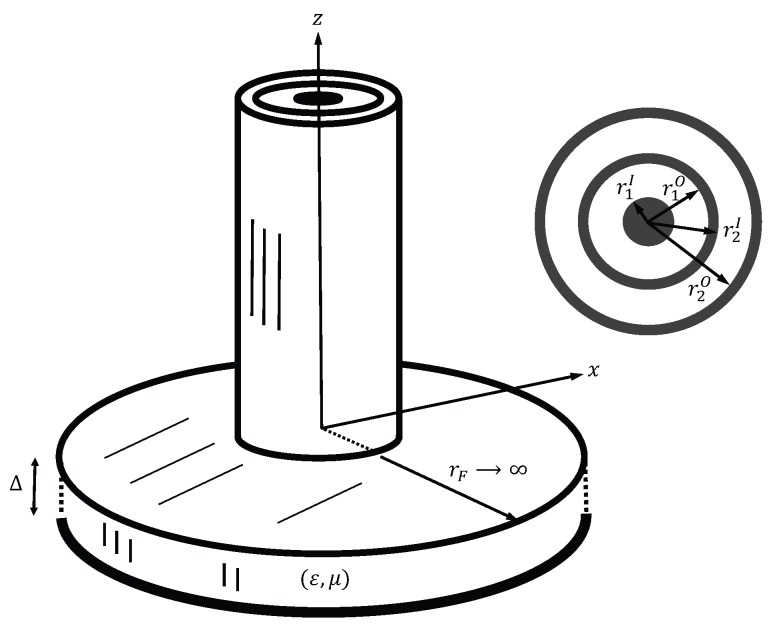
Simple theoretical model of the triaxial applicator.

Because the measured S-parameters are de-embedded to the material’s plane, the theoretical model of the triaxial system is significantly less complicated than the true system. This model is shown in Figure 2. Two concentric air-filled coaxial cables constructed from a perfect electric conductor (PEC) open into a PEC flange that is assumed to be infinite in extent. The inner and outer radii of the inner cable (cable 1) and the outer cable (cable 2) are shown in the figure. The flange is placed against a conductor-backed material sample of permittivity ϵ and permeability μ . The plane of contact, called the material’s plane, defines the positions of the ports at which the material is interrogated by the TEM waves in the cables. These ports are named Port A for cable 1 (the inner cable) and Port B for cable 2 (the outer cable). We note that the port names Port 1 and Port 2 are reserved for the measurement ports for which the network analyzer cables are connected; see Section 4.

A TEM_z_ wave of amplitude $a_{1}$ is assumed incident along the −z direction in cable 1, and a TEM_z_ wave of amplitude $a_{2}$ is assumed incident along the −z direction in cable 2. Interaction with the material sample produces a reflected TEM_z_ wave in cable 1 of amplitude $b_{1}$ traveling in the +z direction and similarly a reflected wave of amplitude $b_{2}$ in cable 2. The desired reflection coefficients at Ports A and B are then given by

(3) $$S_{11}^{\mathrm{THY}}=\frac{b_{1}}{a_{1}}{|}_{a_{2}=0}\;S_{22}^{\mathrm{THY}}=\frac{b_{2}}{a_{2}}{|}_{a_{1}=0}$$

respectively, while the transmission coefficients between Ports A and B are given by

(4) $$S_{21}^{\mathrm{THY}}=\frac{b_{2}}{a_{1}}{|}_{a_{2}=0}=S_{12}^{\mathrm{THY}}=\frac{b_{1}}{a_{2}}{|}_{a_{1}=0}$$

The incident TEM_z_ wave will also generate higher-order TM_z_ modes in each coaxial cable. Thus, the transverse fields in cable *i* ( i=1,2 ) can be written as a modal expansion, and the modal amplitudes may be obtained by solving a magnetic-field integral equation (MFIE) formulated by applying continuity of the transverse fields at the material’s plane (z = 0). A detailed description of the solution to the theoretical problem is given in [22] and is not repeated here for the sake of brevity.

## 4. Calibration Procedure

**Figure 3:**
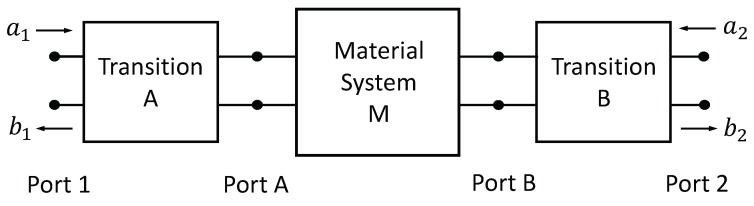
Two-port model of the triaxial system, showing coaxial transitions A and B.

Because the reflection and transmission coefficients at the material’s plane are needed for the material characterization, a calibration procedure is required to convert the measured S-parameters at connection Ports 1 and 2 to the desired S-parameters at Ports A and B. This calibration procedure compensates for the phase shift and losses between the measurement ports and the probe aperture, as well as for the imperfect coupling between the side cable and the outer coaxial section of the applicator. It is assumed that the distance from the connection point of the side cable and Port 2 is sufficiently large such that higher-order modes generated at the connection are of negligible amplitude at Port 2 and may thus be neglected. Similarly, it is assumed that the distance from the connection point to the material’s plane is such that higher-order modes generated at the connection point may be neglected at Port B. Finally, it is assumed that the distance from Port A to Port 1 is such that higher-order modes generated at the material’s plane are negligible at measurement Port 1. Then, only the dominant TEM modes are incident at Ports A and B, and only dominant modes are returned to the measurement Ports 1 and 2; thus the triaxial system may be modeled using a two-port analysis, as shown in Figure 3.

### 4.1. Analysis as Cascaded Two-Port Networks

The triaxial system is modeled as three cascaded two-port networks. The transition network A corresponds to the inner coax of the triaxial system, extending from the measurement Port 1 to the material plane’s Port A. This network includes the propagation effects of the dominant mode in the inner coax and also the mismatch reflection at Port 1 due to the difference in impedance of the measurement system (assumed to be 50 Ω ) and the characteristic impedance of the inner coax $Z_{A}$ (which is slightly less than 50 Ω ). Network B corresponds to the transition between measurement Port 2 on the side cable and the material plane’s Port B. Network B describes the coupling between the side cable and the outer coax of the triaxial system, propagation effects in the side cable and outer coax, and also the mismatch reflection at Port 2 due to the difference in impedance of the 50 Ω measurement system and the characteristic impedance of the side cable (which is the same as the inner coax, $Z_{A}$ ). Finally, network M represents the dominant mode interaction of the inner and outer coaxial cables with the parallel-plate structure at the material’s plane (as described by the theoretical model). Thus, the goal is to de-embed the material plane’s S-parameters, $S_{AA}=S_{11}^{M}$ , $S_{BA}=S_{21}^{M}$ , $S_{AB}=S_{12}^{M}$ , and $S_{BB}=S_{22}^{M}$ , from measurements of $S_{11}$ , $S_{12}$ , $S_{21}$ , and $S_{22}$ . We note that because the material under test is assumed to be isotropic, the system is reciprocal, and therefore $S_{12}=S_{21}$ and $S_{12}^{M}=S_{21}^{M}$ .

### 4.2. Measurement of Transition Network S-Parameters

**Figure 4:**
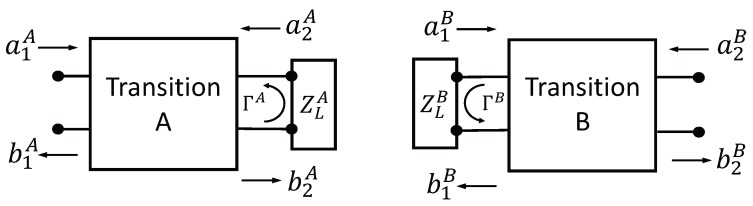
Termination of transition networks with known loads.

Calibration is accomplished by determining the S-parameters of network A ( $S_{11}^{A}$ , $S_{12}^{A}$ , $S_{21}^{A}$ , and $S_{22}^{A}$ ) and those of network B ( $S_{11}^{B}$ , $S_{12}^{B}$ , $S_{21}^{B}$ , and $S_{22}^{B}$ ). Once again, because of reciprocity, $S_{12}^{A}=S_{21}^{A}$ and $S_{12}^{B}=S_{21}^{B}$ . To find the desired S-parameters, each network is considered separately, and the three-short technique is used [24]. We consider first network A, as shown in Figure 4. When a known load $Z_{L}^{A}$ is attached to port 2 of network A, the resulting reflection coefficient $\Gamma^{A}$ is determined through the relationship $a_{2}^{A}=\Gamma^{A}b_{2}^{A}$ . Thus, the network equations are

(5) $$\begin{array}{ccc} b_{1}^{A} & = & S_{11}^{A}a_{1}^{A}+S_{12}^{A}a_{2}^{A}=S_{11}^{A}a_{1}^{A}+S_{12}^{A}\Gamma^{A}b_{2}^{A} \end{array}$$

(6) $$\begin{array}{ccc} b_{2}^{A} & = & S_{21}^{A}a_{1}^{A}+S_{22}^{A}a_{2}^{A}=S_{21}^{A}a_{1}^{A}+S_{22}^{A}\Gamma^{A}b_{2}^{A} \end{array}$$

Solving these equations simultaneously gives

(7) $$\frac{b_{1}^{A}}{a_{1}^{A}}=S_{11}^{A,M}=S_{11}^{A}+\frac{S_{12}^{A}S_{21}^{A}}{1-S_{22}^{A}\Gamma^{A}}\Gamma^{A}$$

Here, $S_{11}^{A,M}$ represents the reflection coefficient measured at port 1 of network A when the load is placed at port 2 of network A. Rearranging gives

(8) $$\frac{1}{\Gamma^{A}}=S_{22}^{A}+\frac{S^{A}}{S_{11}^{A,M}-S_{11}^{A}}.$$

Here

(9) $$S^{A}=S_{12}^{A}S_{21}^{A}$$

is defined, as the S-parameters $S_{12}^{A}$ and $S_{21}^{A}$ always appear as a product.

Now, we assume that the S-parameters $S_{11}^{A,M(1)}$ , $S_{11}^{A,M(2)}$ , and $S_{11}^{A,M(3)}$ are measured, corresponding to three distinct loads with reflection coefficients $\Gamma^{A(1)}$ , $\Gamma^{A(2)}$ , and $\Gamma^{A(3)}$ , respectively. Writing Equation (8) three times and solving the three equations simultaneously gives the desired S-parameters for network A:

(10)S22A=KAΓA(1)−1ΓA(3)KA−1(11)SA=S11A,M(1)−S11A,M(2)1ΓA(2)−1ΓA(1)1ΓA(1)−S22A1ΓA(2)−S22A(12)S11A=S11A,M(1)−SA1ΓA(1)−S22A

Here,

(13) $$K^{A}=\left(\frac{S_{11}^{A,M(1)}-S_{11}^{A,M(2)}}{S_{11}^{A,M(2)}-S_{11}^{A,M(3)}}\right)\left(\frac{\frac{1}{\Gamma^{A(3)}}-\frac{1}{\Gamma^{A(2)}}}{\frac{1}{\Gamma^{A(2)}}-\frac{1}{\Gamma^{A(1)}}}\right)$$

We note that because $S_{12}^{A}=S_{21}^{A}$ , $S_{21}^{A}=\pm \sqrt{S^{A}}$ , where the proper sign is chosen from the expected behavior of the network.

Repeating the above procedure for network B, and noting that the ports for network B are the opposite of those for network A, it is found that

(14)S11B=KBΓB(1)−1ΓB(3)KB−1(15)SB=S11B,M(1)−S11B,M(2)1ΓB(2)−1ΓB(1)1ΓB(1)−S11B1ΓB(2)−S11B(16)S22B=S11B,M(1)−SB1ΓB(1)−S11B

Here,

(17) $$K^{B}=\left(\frac{S_{11}^{B,M(1)}-S_{11}^{B,M(2)}}{S_{11}^{B,M(2)}-S_{11}^{B,M(3)}}\right)\left(\frac{\frac{1}{\Gamma^{B(3)}}-\frac{1}{\Gamma^{B(2)}}}{\frac{1}{\Gamma^{B(2)}}-\frac{1}{\Gamma^{B(1)}}}\right)$$

As with network A, because $S_{12}^{B}=S_{21}^{B}$ , $S_{21}^{B}=\pm \sqrt{S^{B}}$ , where the proper sign is chosen from the expected behavior of the network.

### 4.3. De-Embedding the Material Network S-Parameters

Once the S-parameters for networks A and B are found, the material network M may be de-embedded using matrix inversion. First the S-parameter matrices $\left[S^{A}\right]$ and $\left[S^{B}\right]$ are converted to transmission matrices $\left[T^{A}\right]$ and $\left[T^{B}\right]$ using the general conversion formulas:

(18) $$T_{11}=\frac{S_{21}S_{12}-S_{11}S_{22}}{S_{21}},\;T_{12}=\frac{S_{11}}{S_{21}},\;T_{21}=-\frac{S_{22}}{S_{21}},\;T_{22}=\frac{1}{S_{21}}$$

Next it is assumed that the S-parameters of the triaxial system are measured with a sample in place, giving the overall scattering matrix [S] , which may be converted to the transmission matrix [T] . Then, $\left[T\right]=\left[T^{A}\right]\left[T^{M}\right]\left[T^{B}\right]$ , and thus the de-embedded transmission matrix is

(19) $$\left[T^{M}\right]={\left[T^{A}\right]}^{-1}\left[T\right]{\left[T^{B}\right]}^{-1}$$

Finally, the S-parameters of the de-embedded material’s network may be found by applying the general conversion formulas:

(20) $$S_{11}=\frac{T_{12}}{T_{22}},\;S_{12}=\frac{T_{11}T_{22}-T_{12}T_{21}}{T_{22}},\;S_{21}=\frac{1}{T_{22}},\;S_{22}=-\frac{T_{21}}{T_{22}}$$

### 4.4. The Three-Short Method

Perhaps the simplest loads to use to determine the transition network S-parameters are short circuits. In this implementation, shorting plates are placed along the inner and outer coaxial cables at three different positions relative to the material’s plane. These distances can be positive, in which case the coaxial cables are extended in length, or negative, in which case the shorts are inserted into the cables, thus shortening their lengths.

To determine the reflection coefficient of the shorting plates, it is assumed that a coaxial line of length *d* is attached to either the inner or outer cable and that the characteristic impedance of the attached cable matches that of the cable to which it is attached. If *d* is negative, then the length of the inner or outer cable is shortened by inserting a shorting plug into the respective cable. In any case, the reflection coefficient is computed assuming a perfect reflection at the short:

(21) $$\Gamma =-e^{-2\gamma d}$$

Here γ is the complex propagation constant of the TEM mode of the coaxial cable, given by

(22) $$\gamma =\sqrt{\left(R+j\omega L)(G+j\omega C\right)}$$

where *R* is the resistance per unit length:

(23) $$R=\frac{R_{s}}{2\pi a}\left(1+\frac{a}{b}\right)$$

*L* is the inductance per unit length:

(24) $$L=\frac{\mu_{0}}{2\pi }\left[\ln\left(\frac{b}{a}\right)+\frac{\delta }{2a}\left(1+\frac{a}{b}\right)\right]$$

and *C* is the capacitance per unit length:

(25) $$C=\frac{2\pi \epsilon_{0}}{\ln\left(b/a\right)}$$

In these expressions, *a* is the radius of the inner conductor of the coaxial cable, *b* is the radius of the outer conductor, $\delta =1/\sqrt{\pi f\mu_{0}\sigma }$ is the skin depth of the conductor from which the coaxial cables are constructed, σ is the conductivity of the conductor, and $R_{s}=1/\left(\sigma \delta \right)$ is the surface resistance. Because the prototype considered in this paper uses an air dielectric for all coaxial cables, the conductance per unit length, *G*, is assumed to be zero. We note that when the conductors are imperfect and R≠0 , the characteristic impedance of the coaxial cables is complex with a small imaginary part, as is given by

(26) $$Z_{c}=\sqrt{\frac{R+j\omega L}{G+j\omega C}}$$

### 4.5. Example of Calibration

It is desirable to test the calibration scheme before implementing it with measured data. To this end, the triaxial system shown in Figure 1 was simulated using the commercial High-Frequency Structure Simulator (HFSS) software. The geometry of the structure was identical to that of the prototype described in the next section (except that the length of the center cable was slightly longer and the flange was thicker in the prototype). Both the inner cable and side cable were identical coaxial structures. The center conductor was a solid brass cylinder of diameter 6.25 mm. The outer sheath was a tube with an inner diameter of 13.8 mm and a thickness of 1 mm. The same tube formed the inner conductor of the outer cable. The outer sheath of the outer cable was a tube with an inner diameter of 34.747 mm and thickness of 1 mm. The length of the inner cable was 200 mm, and the length of the side cable was 75 mm; these lengths were sufficient to prevent higher-order modes from appearing at Ports 1 and 2. The flange had a diameter of 178.1 mm and a thickness of 1 mm. The height of the outer cable from the bottom of the flange to the shorting plate was 200 mm, and the height of the inner tube to Port 1 was 200 mm. Finally, the inner conductor of the side cable was connected to the inner conductor of the outer cable 40 mm beneath the shorting plate, a distance determined through trial and error, as discussed below. All parts were constructed using 260 brass with conductivity of $\sigma =1.62\times 10^{7}$ S/m. The characteristic impedance of the inner and side cables varied from 47.52−j0.0318Ω at 750 MHz to 47.51−j0.0159Ω at 3 GHz. The characteristic impedance of the outer cable varied from 47.26−j0.0126Ω at 750 MHz to 47.26−j0.00630Ω at 3 GHz. It was thus fairly safe to consider the characteristic impedances of both cables to be real and frequency-independent.

The triaxial structure shown in Figure 1 was simulated in the HFSS to obtain the S-parameters $S_{11}$ , $S_{21}$ and $S_{22}$ . The material sample was taken to be a MagRAM of thickness 3.175 mm (0.125 in) backed by a brass plate. The material properties of the sample were set at $\epsilon =\left(7.32-j0.00464\right)\epsilon_{0}$ and $\mu =\left(0.576-j0.484\right)\mu_{0}$ . These are the properties of the commercial MagRAM Eccosorb™ FGM-125 (Emerson & Cuming, Geel, Belgium) at 10 GHz [17]. Although the material properties were frequency-dependent and would have different values at lower frequencies, the values quoted were assumed to be frequency-independent and to represent typical MagRAM parameters for the purpose of testing the calibration routine.

**Figure 5:**
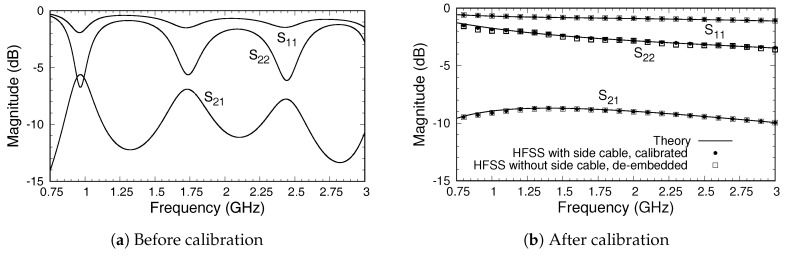
Magnitudes of the S-parameters of the triaxial system before and after calibration. Material sample is representative magnetic radar absorbing material (MagRAM).

Figure 5a,b show magnitudes of the simulated S-parameters for the triaxial system before and after calibration was employed, respectively. We note that the S-parameters were relative to the true port impedances at Ports 1 and 2. The S-parameters could be renormalized to 50 Ω if desired using the method in [25], but as mentioned earlier, issues regarding mismatches at the ports were handled by the calibration procedure. Thus, the S-parameters after calibration were relative to the true port impedances even if measured using a 50 Ω system. The oscillations visible in each S-parameter were primarily due to standing waves established between the side-cable transition and the measurement plane and were eliminated upon calibration. We note that the transmission coefficient varied between about −14 and −6 dB across the measurement band, and thus even before calibration there was significant transmission between the ports and through the sample.

**Figure 6:**
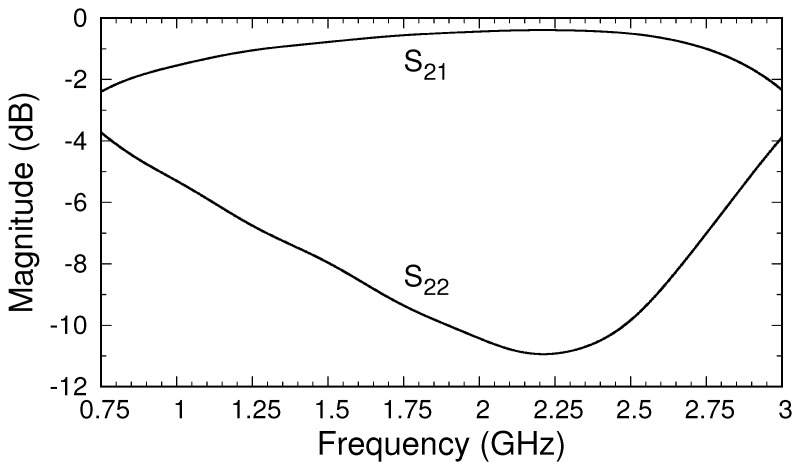
Magnitudes of the S-parameters of transition B. Side-cable placement was based on the behavior of $|S_{21}|$ across the operating band.

The calibration of the triaxial system was accomplished using brass short circuits of lengths 0, −12.5 , and −25 mm. The negative lengths indicate that the shorts extended into the triaxial structure by these distances and corresponded to electrical lengths of 0, λ/8 , and λ/4 at 3 GHz, the highest frequency in the band of interest. Employing the calibration routine described in Section 4, the system’s S-parameters for the transition regions were first found. Those for transition *A*, representing the center coax, correct for the phase shift and slight attenuation of the center cable. The S-parameters of transition *B* correct for the imperfect transmission from Port 2 to Port B and include the effects of reflections and higher-order mode generation at the connection of the center conductor of the side cable and the inner conductor of the outer cable. Figure 6 shows the magnitude of the S-parameters of transition B. The choice of position of the side cable relative to the top of the outer cable was made by examining the S-parameters of transition B for several possible placements. The decision to use a position of 40 mm was based on making $|S_{21}|$ as large and uniform as possible across the operating band of 0.75–3 GHz. The result was a transition with $|S_{21}|≳-2$ dB across the band.

**Figure 7:**
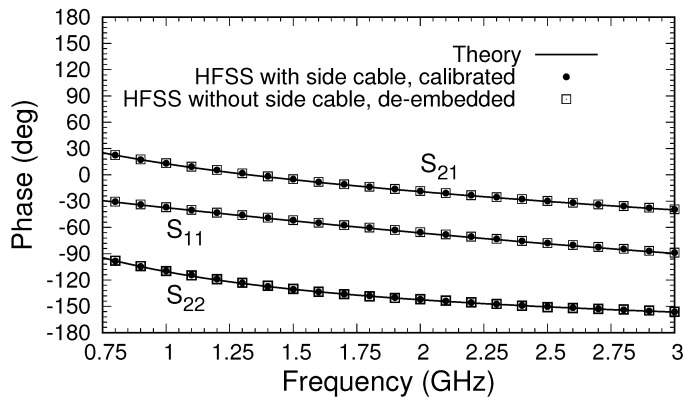
Phases of the S-parameters of the triaxial system after calibration.

The magnitudes of the S-parameters after calibration, shown in Figure 5b, are compared to those found using the theoretical approach described in [22], which produces S-parameters directly at the material’s plane (Ports A and B). Because the theoretical model uses an expansion of the fields in terms of coaxial cable modes, a choice of the number of modes to use must be made. To obtain the results shown, the S-parameters were computed using 5, 10, and 20 modes, and the results were extrapolated quadratically to an infinite number of modes [26]. A comparison between the S-parameters from the HFSS after calibration and those obtained from theory is excellent, particularly because the theoretical model assumes the flange and sample are of infinite extent and that the flange and conductor backing are perfect electric conductors. As a further test of the accuracy of the calibrated values, HFSS simulations of the simple model of the triaxial system shown in Figure 2 were made. The S-parameters were computed at the inputs to the inner and outer cables, and the de-embedding function in the HFSS was used to transfer the S-parameters to Ports A and B (at the material’s plane). The results, shown in Figure 5b, are very close to those found using the three-short calibration method with the full triaxial system but are not an exact match. The difference is attributed to inaccuracies in the HFSS calculations and to the possible existence of higher-order modes of very small but nonzero amplitudes at the measurement ports. In any case, the results do support using the three-short method to calibrate measured data from the triaxial system, suggesting that these results should match the theory well enough to provide robust data for material characterization. Comparison of the phases from the three approaches, shown in Figure 7, supports this conclusion.

## 5. Design and Fabrication of a Prototype Triaxial Probe System

**Figure 8:**
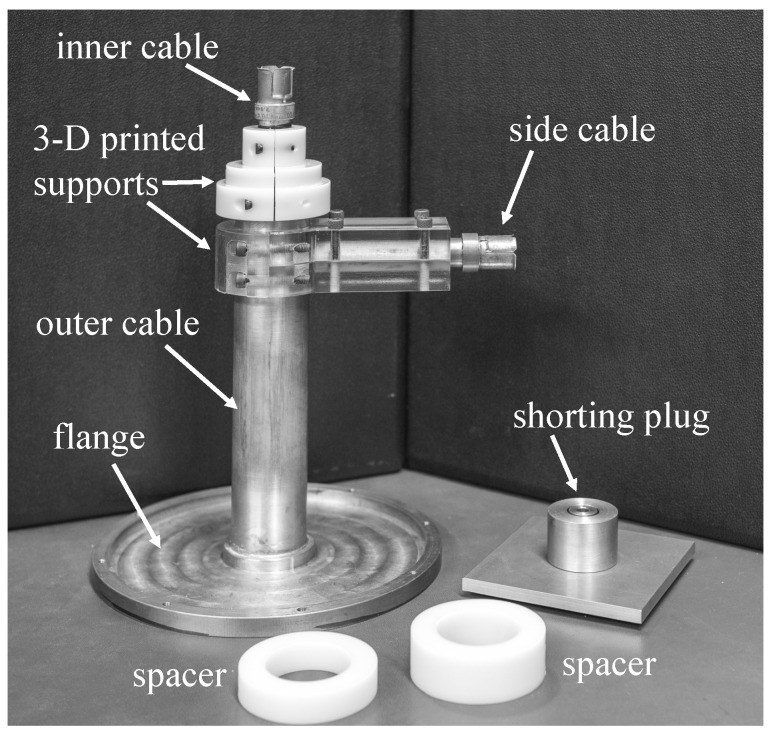
Fabricated triaxial applicator. Also shown are the calibration shorting plug and its spacers.

A prototype triaxial probe system was designed to operate in the band 0.75–3 GHz using mostly parts on hand. The inner and side cables were constructed using prefabricated General Radio (GR) airlines and were terminated in GR-874 50 Ω connectors [27,28]. The diameter and tube thickness were identical to those in the simulations described in Section 4.5, and thus the characteristic impedance of these cables was slightly less than 50 Ω . However, the length of the inner cable for the prototype was slightly longer at 235 mm to allow for attachment of the GR connector. The outer conductor of the outer cable was fabricated using standard brass tubing, again with dimensions given in Section 4.5. The inner cable was supported within the tube using thin Teflon spacers, with holes drilled to minimize reflections. The outer cable was soldered to a brass flange of radius $r_{F}=87.4$ mm and thickness 5 mm. The final fabricated prototype is shown in Figure 8, along with the shorting plug and its spacers.

The side cable was inserted through a hole in the outer conductor of the outer cable and was press-fit into place. The inner conductor of the side cable was press-fit against the inner conductor of the outer cable, and the electrical connection was enhanced using silver paste ( $\sigma =5.88\times 10^{3}$ S/m). Finally, a brass cap was press-fit on top of the outer cable. All the press fit connections were kept in place by using three-dimensional (3D) printed plastic clamps.

## 6. Measured Results

The triaxial system was calibrated using the three-short method. A shorting plug was constructed from brass with a height of 25 mm, as shown in Figure 8. Two plastic rings, one of thickness 20 mm and one of thickness 12.5 mm, were 3D printed to be used as spacers. Thus, the three calibration standards were shorts located at positions d=−5 , −12.5 , −25 mm. These distances were about λ/8 apart at 3 GHz. Using a total span of λ/4 prevented issues involving phase ambiguity at the highest frequency of interest.

Some difficulties were encountered with the constructed shorting plug. The plug did not fit snugly into the outer cable, and a small gap allowed the plug to rock slightly when inserted by 5 mm. The gap also allowed the fields to escape at certain frequencies, causing $S_{11}$ for the transition network B to drop out by one or two decibels. This was most prominent below 1 GHz and around 2.25 GHz. The result was that the de-embedded parameters showed moderate deviations near these frequencies. The results could be improved somewhat by setting the magnitude to 0 dB but keeping the phase intact. The longer shorts did not produce these negative effects, and thus a possible solution is to use a longer shorting plug that still has positions about λ/8 apart at the highest frequency of interest. It would be necessary to ensure that there were no higher-order coaxial modes present at the new shorting positions. If there were, then the triaxial system would need to be lengthened. This will be explored in the future when an updated applicator is designed and constructed.

**Figure 9:**
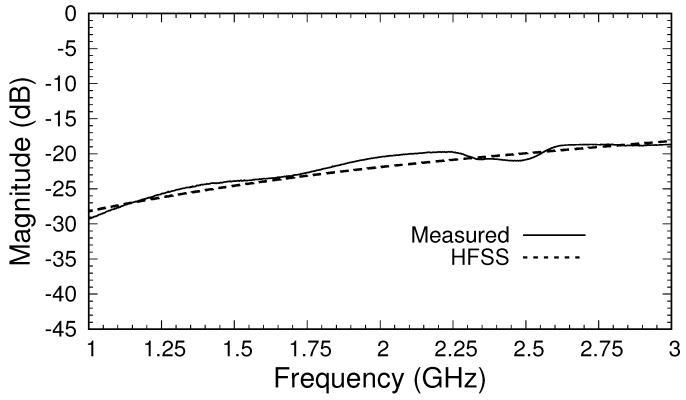
Magnitude of measured $S_{21}$ after calibration for the triaxial system in air.

**Figure 10:**
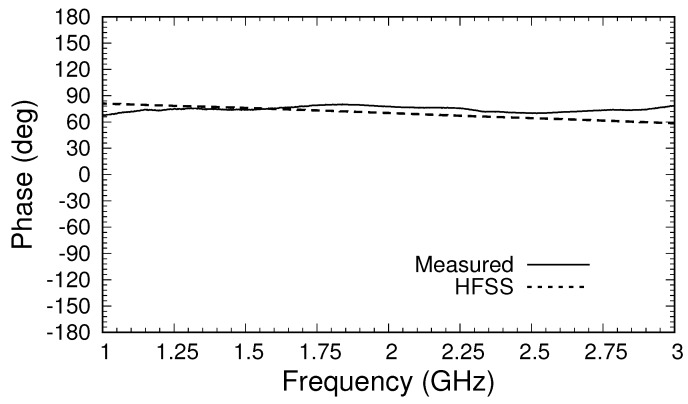
Phase of measured $S_{21}$ after calibration for the triaxial system in air.

All measurements were made using an Agilent N9917A VNA with a 10 kHz intermediate frequency bandwidth and 16 averages. As a simple test of the performance of the calibrated triaxial system, $S_{21}$ was measured without a sample present (with the applicator open to air). The magnitude and phase of this transmission coefficient are shown in Figure 9 and Figure 10. Values at frequencies below 1 GHz are not shown because of the deviation caused by the loose plug in the outer cable. The effect of the loose plug is also seen around 2.25 GHz, where both the magnitude and phase differed from that predicted by the HFSS. Overall, the magnitude agreed with simulations to within about 1 dB over the measurement band, and the phase agreed to within about 5 degrees. This agreement was likely insufficiently accurate to allow for good material parameter extraction, and thus future systems will need to employ an improved shorting system for calibration. Nevertheless, the results show that the calibration and measurement processes are valid.

**Figure 11:**
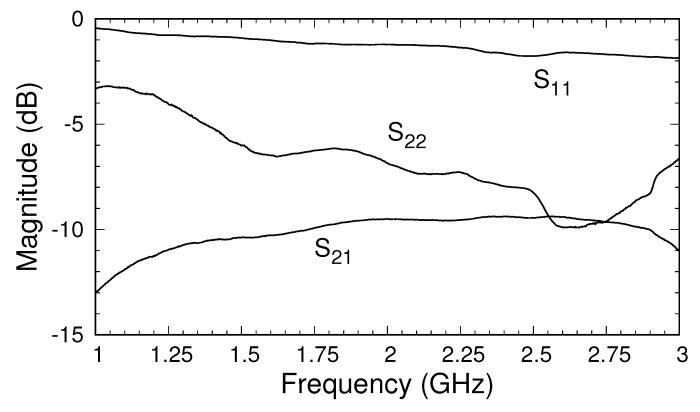
Magnitude of measured S-parameters after calibration for a sample of FGM-125.

**Figure 12:**
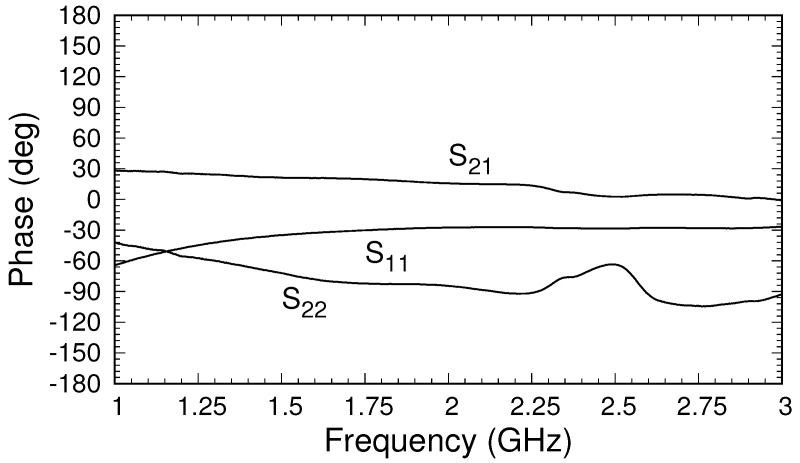
Phase of measured S-parameters after calibration for a sample of FGM-125.

To provide insight into typical material measurements, a sample of FGM-125 was measured, and the results are shown in Figure 11 and Figure 12. The sample was placed between the flange of the triaxial applicator and an aluminum backplate and was held in place using clamps. Both the magnitude and phase of the measured S-parameters were quite similar to the simulated and theoretical values shown in Figure 5b and Figure 7. We recall that the simulated data were generated using material parameters valid at 10 GHz, and thus perfect agreement was not expected. In fact, while the permittivity of FGM-125 was fairly independent of frequency, the permeability varied significantly below a few gigahertz. The values of $S_{22}$ fluctuated more than those of $S_{11}$ and $S_{21}$ . This was due to calibration issues arising from the imperfect short in the outer cable, as discussed above. It is expected that an updated shorting system will greatly improve these results. Regardless, the measurements show that the proposed triaxial system produces significant coupling between the inner and outer cables, which should provide excellent data for parameter extraction once the construction of the system is optimized.

## 7. Discussion

The triaxial system described in this paper shows great promise for use in material characterization. A design of a prototype system has been presented and a calibration technique devised and validated using simulations and theory. Measurements presented using the outlined calibration technique demonstrate that the system produces significant coupling between the measurement ports, which is important for accurate material characterization. Some issues with the current prototype have been identified, primarily with regard to the shorting plug used for calibration. Future work includes improving the calibration standard either by lengthening the plug or including fingers to improve electrical contact, or both. A smaller system for use at higher frequencies is also planned. Finally, a parameter extraction routine will be implemented using data measured with the system and results compared to those obtained using other methods.

## Abbreviations

The following abbreviations are used in this manuscript:

GR	General Radio
HFSS	High-Frequency Structure Simulator
MagRAM	Magnetic radar absorbing material
MFIE	Magnetic-field integral equation
TEM	Transverse electromagnetic
TM	Transverse magnetic
VNA	Vector network analyzer
